# Improving adoption of technologies and interventions for increasing supply of quality livestock feed in low- and middle-income countries

**DOI:** 10.1016/j.gfs.2020.100372

**Published:** 2020-09

**Authors:** Mulubrhan Balehegn, Alan Duncan, Adugna Tolera, Augustine A. Ayantunde, Salissou Issa, Moctar Karimou, Nouhoun Zampaligré, Kiema André, Isidore Gnanda, Padmakumar Varijakshapanicker, Ermias Kebreab, Jose Dubeux, Kenneth Boote, Muluneh Minta, Fekede Feyissa, Adegbola T. Adesogan

**Affiliations:** a Feed the Future Innovation Lab for Livestock Systems, University of Florida, 2250 Shealy Drive, Bldg 459, Room 125, Gainesville, Florida, USA; b Department of Animal, Rnageland and Wildlife Sciences, Mekelle University, Ethiopia; c International Livestock Research Institute, Ethiopia; d School of Animal and Range Sciences, Hawassa University, Hawassa, Ethiopia; e International Livestock Research Institute, Burkina Faso; f L'Institut National de la Recherche Agronomique du Niger/National Institute of Agricultural Research, Niger; g Mercy Corps, Niger; h Institut de l’Environnement et de Recherches Agricoles, Burkina Faso; i International Livestock Research Institute, India; j University of California Davis, USA; k Department of Agronomy, Institute of Food and Agricultural Sciences, University of Florida, USA; l Department of Animal Sciences, Institute of Food and Agricultural Sciences, 2250 Shealy Drive, Bldg 459, Room 125, University of Florida, USA; m Ethiopian Institute of Agricultural Research, Ethiopia

**Keywords:** Feed, Feed improvement, Smallholder livestock producer, Technology adoption

## Abstract

The global increase in the demand for and production of animal-source foods (four-to five-fold increase between 1960 and 2015), which has been mostly concentrated in low- and middle-income countries (LMIC), provides smallholder livestock producers with an opportunity for improving their livelihoods and food and nutrition security. However, across livestock production systems in many LMIC, limited supplies and high cost of good quality feed severely constrains exploitation of this opportunity. In many of such countries, feeds and feeding-related issues are often ranked as the primary constraint to livestock production and increased consumption of animal-source foods. Here we review the complex biophysical, socio-economic and technological challenges related to improving quality feed supply and the reasons for generally low adoption of apparently proven feed enhancement technologies. We describe also successful interventions and conclude by recommending strategies for improving quality feed supply in LMIC that account for and overcome the prevailing challenges.

## Highlights

• Livestock production in developing countries is constrained by limited supply of quality feeds.
• There are many technologies that can improve supply of quality feed for small holder producers.
• Complex set of bio-physical and socio-economic factors limit the adoption of technologies and thus limit the improvement in supply of quality feed.
• The failures of adoption of feed improvement technologies result from systemic constraints and inadequate attention to sociocultural and economic factors.
• Success is often driven either by market incentives and or an innovative affordable approach that provides solutions to multiple problems.

## 1 Introduction

Livestock are an important livelihood source for over 900 million small scale producers in low-and middle-income countries (LMIC), a source of nutrient-dense animal source foods (ASF) and income for members of such households (Dolberg, 2001). Livestock production systems in LMICs range from extensive mobile pastoral to semi-intensive urban and peri-urban small-scale production systems.

Demand for livestock products is increasing worldwide, particularly in LMIC, fueled by population growth, urbanization, and rising incomes (Alexandratos and Bruinsma, 2012). Per capita consumption of ASF increased four to five-fold from the early 1960s to 2015 (Ritchie, and Roser, 2017), with three fourths of this growth coming from LMIC (Delgado, 2003; Delgado, 2005). By 2050, consumers in LMIC will demand 107 million tons more meat and 5.5 million tons more milk than they did in 2005/2007 (Alexandratos and Bruinsma, 2012). The increase in demand pertains to both quality and quantity, especially as incomes rise from USD 2 to 10 per day, particularly among urban consumers who purchase livestock products from supermarkets (Thornton et al., 2007).

Though the livestock revolution can be an important opportunity for increasing income for small scale producers in LMIC, various constraints limit their ability to take advantage of it. Among many systematic, bio-physical and socio-cultural problems, limited access to quality feed is a major challenge across all production systems in LMIC (Owen et al., 2012).

Feed affects livestock productivity, profitability, environmental impact, human food and nutrition security, animal welfare and ethics, and animal and human health (Makkar, 2016a). Financially, feed costs account for up to 70% of the total variable costs of livestock production and may reach 90% in more intensive systems (Makkar, 2016a). The global value of purchased compound feed relative to total animal output is about 30% on average for all production systems, and is 10% for cattle, 40% for pigs and 80% for poultry (FEFAC, 2016). Good quality feed improves livestock productivity, resulting in lower age at first calving and shorter inter-calving interval, thus increasing productive life and profitability (Linde et al., 2002). Proper feeding improves animal immunity (Vighi et al., 2008), health, welfare, and reproductive performance; enables higher productivity under a given management regimen (Absalón-Medina et al., 2012) and contributes to environmental sustainability by converting energy and nutrients from land that is unusable by humans into highly nutritious food. Much of the feed consumed from livestock is derived from such areas or inedible agro-industrial byproducts. In fact, about 86% of the feed globally consumed by livestock is not edible by humans (Mottet et al., 2017). Properly implemented forage-based systems including silvopastoral systems can reduce emissions from livestock (Rao et al., 2015). Proper feeding can reduce the methane emissions from livestock farming by increasing the ratio of feed used for production to that used for maintenance, also known as the maintenance dilution effect (Garg et al., 2013). This is critically important since feed (production, processing), and enteric fermentation contribute to 45% and 39%, respectively, of the total emissions from livestock production (Steinfeld et al., 2006).

Lack of availability and access to quality feed continues to be the most important limitation to livestock production in LMIC. For instance, in six African and Asian focus countries of the Feed the Future innovation Lab for Livestock Systems, feeds and feeding-related issues were ranked consistently as the primary constraint to livestock production and consumption of ASF (ME, 2018). There are, important market opportunities and drivers for improving the supply of quality feed in developing countries. These include population growth, urbanization and the rising incomes, and these demand factors provide a strong rationale for improving the supply of quality feed in LMIC. The demand is fueled by increasing human population and per capita income and the concomitant increase in demand for ASFs (Steinfeld et al., 2006). Such increases in production and demand for ASF have resulted in a burgeoning of entrepreneurs engaged in livestock input production and supply, including in the feed industry. For instance, between 2009 and 2016, compound feed production increased by 14.1% in USA, 18.9% in Brazil, 24.5% in Europe, 74.8% in China, and by 106.3% in the Middle East and Africa, indicating that increases were greater in areas that are growing quickly economically and demographically (FEFAC, 2016).

The increase in demand for livestock feed has led to introduction of a diverse set of technological solutions that promise to increase production of quality feed with limited resources. These generally can be categorized into five groups namely: 1. **Feed productivity improvement,** aimed at improving biomass production or availability of feed; 2. **Feed quality enhancement,** focused on improving nutritional value, palatability, intake and digestibility of low quality feeds; 3. **Feed quality maintenance or preservation,** aimed at preserving the nutritional quality of feeds during storage for off-season feeding; and 4. **Enhancement of the nutritional status of animals,** through supplementation of animal diets with highly nutritious ingredients that supply critical nutrients or enhance digestion and assimilation of feed. In addition, further technological development focuses on 5. **Analytical and operational technologies,** such as improvements to feed quality analysis, quality control, marketing, packaging, transporting and feeding. Table 1 provides descriptions of various technologies in these categories. Many LMIC are developing and implementing a combination of these different categories of technologies for improving the supply of quality feed. Some of the most commonly applied technologies include introduction of improved forage varieties, enhancing the quality of existing low quality crop residues and roughages, improving the production and utilization of processed concentrates and agro-industrial by-products, and encouraging involvement of the private sector in supplying inputs for or in processing, preserving or marketing feeds. In addition, efforts are focusing on building capacity for proper feeding and nutrition, quality control and standardization of feed quality, and relating prices to quality, through trainings and awareness creation (Shapiro et al., 2015, 2017). Among these, the Livestock Systems Innovation Lab is engaged in conducting feed landscape analyses to document existing feed types, prices, availability, accessibility and quantities, which will lead to development of or updating of feed databases/feed composition tables; testing of new planted and preserved forage varieties on station, as well as on farm to incentivize adoption by demonstrating potential animal productivity returns; determining nutrient requirements and methane emissions of indigenous small ruminants and cattle; developing the capacity to use near infrared reflectance spectroscopy for feed analysis and developing communities of practice to sustain the use of the systems; developing and deploying ration formulation apps/software for matching nutrient needs and requirements of indigenous and crossbred livestock; and examining effects of strategic supplementation on the performance of livestock.

**Table 1:** Practical feed improvement technologies for smallholder livestock production systems and their expected or observed impacts.

Category	Technology	Production system^a^	Description of technologies	Observed or expected impact	References
Improving feed availability/productivity	Improved forage plants	Small holder mixed crop livestock, semi intensive and intensive	Introducing higher yielding and higher-quality forage species including legumes	Increase forage availability and or nutritive value, reduce seasonal fluctuation in availability	Foster et al., 2009; Mengistu et al., 2017
	Conservation-based forage development	Small holder mixed crop-livestock, pastoral	Introduction of forage plants in natural resource conservation structures such as gullies, terraces, etc., which serve a as source of feed, while reinforcing soil and water conservation	Protect soil loss and land degradation while improving feed availability	Mengistu et al., 2017
	Silvopastures /agro-forestry	Small holder mixed crop-livestock, pastoral, semi intensive peri urban	Using pasture, farmlands and degraded areas for growing trees that synergistically impact pasture productivity	Improved fodder biomass productivity by up to 500% compared to conventional fodder tree growing strategies	Balehegn et al., 2014b
	Food-feed crop integration	Small holder mixed crop-livestock	Intercropping or alley farming to exploit synergies in pest protection and soil and water conservation, while improving availability of forage	Improved soil fertility, reduced pest load to food crops, while improving feed availability.	Lenné et al., 2003
	Protected grazing (exclosures, zero-grazing, cut and carry, rotational grazing, deferred grazing	Small holder mixed crop-livestock, pastoral, semi intensive peri urban	Protection or prescribed grazing on range and grazing lands to protect degraded areas and allow for natural regeneration of forage and improvement of forage production	Improved grazing land productivity, forage biomass, quality of forage produced and reduce grazing land degradation	Yayneshet et al., 2009
	Protected agriculture e.g. hydroponics, green house forage production	Semi-intensive urban and peri-urban	Producing forage under protected conditions in areas and localities where conventional way of production is not possible or ineffective.	Enable forage production in small areas of land or in soilless agriculture. Improved availability of green fodder	Masud and Bhowmik, 2018
	Use of underutilized locally available feed resources	All systems	The use of underutilized locally available feed resources including indigenous fodder species, local brewery residues, etc.	Improved feed availability, reduced need for commercial concentrates, improved farm profitability	Balehegn et al., 2014a
	Improved human-food-waste processing	Intensive urban and peri-urban	The use of affordable drying, cleaning, sorting and processing technologies that enable safe use of human food-waste for livestock feeding	Increased supply of feed in areas where there is resource limitation for growing forage	Makkar, 2016b
	Agronomic interventions on cultivated pastures	All	These include the use of recommendations on sowing rates, spacing, species mixing and association, seed treatment, weed and pest control, irrigation land preparation, shading, control of water logging	Improved forage yields	Nitis, 1999
Enhancing feed quality	Chemical treatment of crop residues	Intensive commercial, semi-intensive urban and peri-urban, small holder mixed crop-livestock	Involves treating crop residues with urea and spraying or soaking in dilute acid and alkaline solutions, etc.	Improved crude protein content (with urea treatment); improved intake and digestibility of crop residues	Sarnklong et al., 2010
	Biological treatment of crop residues	Intensive commercial, semi-intensive urban and peri-urban, small holder mixed crop-livestock	Involves treating crop residues with enzymes, bacterial inoculants or white /brown rot fungi	Improved digestibility and intake of crop residues	Li et al., 2010
	Reducing particle size of crop residues	Intensive commercial, semi-intensive urban and peri-urban, small holder mixed crop-livestock	Chopping and grinding crop residues	Improves intake by animals, reduces bulkiness	Hamed and Elimam, 2009
	Fertilization of crops	Intensive commercial, small holder mixed crop-livestock	Applying fertilizers to improve the nutrient content (mainly CP) of crop residues	Fertilization of crops improves quality (improve CP and digestibility and reduce crude fiber) of crop residues as livestock feed resulting in up to 40% greater milk production	Reddy et al., 2003; Haileslassie et al., 2013
	Forage crop breeding	Intensive commercial, small holder mixed crop-livestock	Selective breeding of forages for developing high yielding and better-quality forage accessions	Improved biomass productivity, feed quality and thus improved livestock productivity	den Hartog and Sijtsma, 2013
Maintaining or conserving feed quality	Correct timing of forage harvesting	Intensive commercial, small holder mixed crop-livestock	Harvesting forages when the nutritional content of the forage is at optimal or when both nutritional value and biomass yield are optimal	Improved intake, digestibility and livestock productivity	Makkar, 2016b
	Silage making	Intensive commercial, semi intensive peri urban	Storing fresh fodder under anaerobic conditions to preserve the quality	Conserved fodder with minimal energy and nutrient loss and spoilage	Titterton and Bareeba, 2000
	Hay making	Intensive commercial, semi-intensive urban and peri-urban, small holder mixed crop-livestock	Reducing loss of nutrients from green fodder by drying	Conserved fodder with minimal energy and nutrient loss and spoilage	Klinner and Shepperson, 1975
	Using preservatives	Intensive commercial, semi-intensive urban and peri-urban,	Using microbes or chemicals that inhibit spoilage organisms and preserve the quality of fresh fodder	Conserved fodder with minimal energy and nutrient loss and spoilage	Wang et al., 2018
Improve the nutritional status of animals	Balanced and or phased rationing or ration formulation	Intensive commercial, semi-intensive urban and peri-urban, small holder mixed crop-livestock	Feeding a balanced ration formulated to meet the nutrient requirements of the animal or targeting rations to animals at specific levels of performance	Improved milk yield by 2-14%, Improve net daily income 10–15%; reduced emission of greenhouse gases by 15-20%	Garg et al., 2013
	Supplementation with concentrates	All systems	Supplementing low quality basal diets of animals with nutritious concentrates	Improved intake, digestibility, body weight gain, and milk yield	Selemani and Eik, 2016
	Supplementation with multi-nutrient blocks	Semi-intensive urban and peri-urban, small holder mixed crop-livestock	Providing animals on low quality basal diets multi-nutrient blocks that provide needed supplementary nutrients	Enabled production of the same amount of milk when 50% less green fodder and 30% less protein supplement was fed; Improved feed intake and protein supply, increased milk yield by 1–1.5 l per day and enhanced reproductive performance in cattle	Makkar et al., 2007
	Supplementation with feed additives	Intensive commercial, semi-intensive urban and peri-urban	Enzymes, probiotics, yeast and other products that are added to feeds to help improve the ability of an animal to digest and assimilate feeds	Improved feed intake, digestion and performance	Ramirez, 2014
Analytical and operational technologies	Near Infrared Reflectance Spectroscopy (NIRS)	Intensive commercial, semi-intensive urban and peri-urban	The use of NIRS technology that enables a quick and affordable assessment of the nutritional quality of various types of feeds without reagents	Improved efficiency and cost effectiveness of feed analysis; may be used to enhance feed marketing	Modroño et al., 2017
	User friendly ration formulation tools	Intensive commercial, semi-intensive urban and peri-urban, small holder mixed crop-livestock	The development and use of simple ration formulation tools such as Excel or mobile phone applications that enable farmers to formulate effective rations based on locally available feed resources	Make ration formulation easier and facilitate its adoption	Chakeredza et al., 2008
	Livestock/feed management applications	Intensive commercial, semi-intensive urban and peri-urban	These are user friendly mobile phone-based applications to monitor, track and analyze feed consumed, produced, wasted, etc.	Help improve livestock farm profitability	Hwang et al., 2011

a These are the most dominant production systems using the specified technology, but most technologies can be adapted for other production systems.

Despite the availability and validation of various technologies that can increase feed quality and supply in diverse agroecological and production settings in LMICs, limited supply of quality livestock feed continues to be a major constraint to livestock production. This is because of low adoption of such technologies by smallholder producers who dominate farming in LMIC (Owen et al., 2012). This inadequate supply of quality feed perpetuates global food insecurity directly by limiting livestock productivity. The resulting limited supply and high cost of livestock products, which is partly due to the high costs of feeds, indirectly contributes to nutrition insecurity due to reduced availability, affordability and accessibility of ASF. Low ASF consumption reduces intake of critical nutrients, which are lacking or less bioavailable in the plant foods that dominate diets of the poor in LMIC. Hence, low ASF consumption contributes to malnutrition or stunting and the associated reductions in growth, health, and cognitive development, of children, particularly infants in the first 1000 days of life (Martorell, 2017). Childhood stunting of the workforce in LMIC has also been associated with reduced earning capacity and gross domestic product of nations (Prendergast and Humphrey, 2014). Consequently, inadequate quality feed supply directly and indirectly contributes to global food and nutrition insecurity and the attendant problems.

These severe effects of inadequate quality feed supply described above call for a revision of the approach of introducing and promoting feed improvement technologies in LMIC. Traditionally, the focus has been on validation of the efficacy of such technologies but as much focus should be given to adoption and scaling them in the complex socioeconomic settings in LMIC. In addition, better targeting of specific technologies to appropriate farming systems is needed. This review contributes to the existing literature on feed resources by characterizing the complex biophysical, socio-economic and technological challenges related to improving quality feed supply and the reasons for generally low adoption of apparently proven feed enhancement technologies. We also describe successful interventions and recommend strategies for improving quality feed supply in LMIC that account for and overcome the prevailing challenges.

### 1.1 Challenges of improving the quantity, quality, supply and utilization of feeds by livestock

The challenges of improving feed quality and quantity in LMIC are related to a complex range of biophysical and socio-economic constraints. In many countries, the most significant and prevalent challenges are associated with the depletion of the resource-base for feed production such as through over grazing and land mismanagement (Shiferaw, 2011), and the inherently low quality of common feed resources such as crop-residues and natural pasture. Low quality crop-residues and natural pasture are the main source of feed in LMIC, for instance they account for 95% of the feed biomass in Ethiopia (FAO, 2018). Commonly introduced strategies to alleviate these problems, such as introduction of improved forages, improving crop residue quality, supplementary feeding, zero grazing and grazing land rehabilitation, have not been very successful due to low adoption by livestock producers (Gebremedhin et al., 2003a). For example, in Ethiopia, planted forages make up only less than 1% of cultivated land (Mekasha et al., 2014), despite more than 50 years of testing forage varieties on experimental sites all over the country (ME, 2018). Similarly, the use of urea treatment of straw, an effective technology in experimental stations, is rarely sustained beyond the life of funded projects, partly because economic and sociocultural issues are not sufficiently addressed (Owen et al., 2012). A possible exception is China where government support for the technology has been strong.

Adoption of livestock feed interventions in LMIC is complex and often technically attractive interventions that are effective in other countries are not readily taken up by smallholder farmers in LMIC (Jackson, 2009). Constraints to adoption of technologies are often context-specific and may involve conditions over which local farmers have no control (Sterk et al., 2013). The diversity in production systems in LMIC also limits adoption by making it difficult to develop technologies that fit each system (Table 2; Reece and Sumberg, 2003). Additional constraints include limited and underdeveloped extension services (Davis, 2008); limited financial support to farmers (Adjognon et al., 2017); and limited market incentives that make investment in technology worthwhile (Jenkins and Miklyaev, 2014).

**Table 2:** Some constraints to adopting technologies for improving feed quality and quantity in low- and middle-income countries.

Category	Technology	Constraints for adoption	References
Feed productivity improvement	Grazing management	Adherence to free grazing, large herd sizes and numbers, resistance to destocking and breed replacement, lack of know how or potential benefits	Gebreyohannes and Hailemariam, 2011a; Cao et al., 2013
	Fertilization of degraded pasturelands	Fertilizers may be too expensive compared to the output obtained; recurrent drought situations may result in reduced yield despite application of fertilizers	ILRI-LIVES, 2016
	Exclosures	Conflicts in sharing forage produced in communal exclosures. Produce insufficient forage to be sustainable	Gebremedhin et al., 2003b; Gebregziabher et al., 2017
	Introducing improved forages	Low yields on smallholder farmer's fields due to lack of quality seeds, land, water, fertilizer, and technical knowledge; limited extension support	Sullivan, 2001; Mekoya et al., 2008
	Multipurpose fodder trees	Most are not adapted to local socio-ecological settings. Farmers lack the requisite technical knowledge in planting, harvesting, utilization, etc.	Franzel et al., 2014
	Forage seed production	Forage seeds are expensive, may be low yielding or poorly adapted to smallholder farmer's environments	Duncan et al., 2011
Feed quality enhancement	Crop residue treatment	Lack of knowledge, labor and capital	Owen et al., 2012
Enhancement of the nutritional status of animals	Supplementation with concentrates	Unaffordable by small holder farmers. Higher cost of feed coupled with lower price of animal produce makes concentrate supplementation unprofitable.	Jenkins and Miklyaev, 2014; Lukuyu et al., 2011
	Introducing multi-nutrient blocks	Resource and labor intensive. Ingredients are usually unavailable and unaffordable to smallholder farmers	Sansoucy, 1995

Sourcing adequate feed for livestock may require land that is used for production of cash crops, and this partly explains poor uptake of introduced forages in some LMIC (Romney et al., 2003). Labor is a further constraint as farmers are reluctant to invest in labor-demanding feed technologies with uncertain returns. For instance, the labor demand for cutting, drying and storing of hay may coincide with a time of the year when such labor could be used more profitably (Coppock, 1991). Lack of access to capital for equipment is another constraint; for instance, silage production adoption is hindered by both the high cost of silage equipment and the labor-intensiveness of manual silage making. The scale at which smallholders in LMIC operate is also an important systemic constraint. The small number of animals on smallholder farms in most LMIC (often less than 10) militates against investment in technologies with small returns. A cost-benefit analysis of fattening small ruminants using concentrate feeds in Ethiopia revealed that the cost of feed was too high relative to the profit (Jenkins and Miklyaev, 2014). Fertilization of degraded pasturelands in Tigray, Ethiopia has resulted in a 4.7-fold increase in forage yield and tripling of harvesting frequency, but the technology was not adopted, because inorganic fertilizers are too expensive and do not help during droughts (ILRI-LIVES, 2016). Similarly, urea treatment was mostly successful when implemented by larger farms in India rather than among small-scale producers (Owen et al., 2012).

Besides these systemic constraints, many feed improvement technologies do not fully address a major issue, which is inadequate yield of quality forage. For example, forage legumes have high nutritive value, but lower biomass yields than low quality forage grasses. Therefore, they may not be appreciated and adopted when availability of biomass is the major need. A study of fodder tree preference in Ethiopia revealed that nutritive value is just one among more than twenty criteria that farmers and pastoralists use to choose what to plant (Balehegn et al., 2015). In fact, many farmers in LMIC do not really understand the concept of nutritive value, and rather focus more on providing bulky feeds that promote gut fill but lack critical nutrients. Rumen or gut-fill was the fourth most important criteria, out of 22 local criteria, used by farmers and pastoralists in Ethiopia to choose which fodder tree to plant (Balehegn et al., 2015). This was also evident from a discussion with small-scale farmers in Niger and an ongoing study by the Feed the Future Innovation Lab for Livestock Systems in Burkina Faso, which noted that farmers feed their animals to fill their guts rather than to satisfy specific nutrient requirements. Clearly, a significant need exists for researchers and extensionists to raise awareness about the importance of forage quality among farmers and pastoralists in LMIC.

Institutional and policy level barriers also limit the adoption of technically superior technologies. Conventional adoption studies have tended to focus on identifying household characteristics that predispose to technology adoption (Gebremedhin et al., 2003a). These studies often rightly conclude that farmer characteristics such as level of education or extent of contact with extension services are important determinants of adoption, but they miss the larger questions around higher-level barriers to change in the livestock feed sector in LMIC. In addition to the biophysical, household demographics and farm level factors, it is important to account for the importance of value chain level or institutional factors and regional or national policy issues that hinder technology adoption, entrepreneurship and commercialization (Klerkx et al., 2010).

Adoption is also limited because interventions fail to adequately account for sociocultural factors that are important to farmers. Smallholder farmers lack the resources and sometimes the incentives to adopt many introduced technologies. Many rural livestock producers, particularly pastoralists, have other priorities such as keeping livestock as a status symbol. Rather than feeding their animals to grow faster, they focus on maximizing the number of animals, even at the expense of productivity, thus they are not interested in feed improvement technologies. In addition, shortage of inputs, weak input and output markets and poor interaction among value chain actors, makes feed expenses too high, limiting the uptake of feed technologies (Kebebe, 2019). In Tanzania a forage chopper thought to be suitable for both sexes (because it reduces labor) was not adopted by women because of gender dynamics within the farmer groups (Fischer et al., 2017). This was because men tended to have greater access to the equipment because they claimed women had “lower technical skills”. Forage varieties that showed promise in on-station research sometimes did not show the same promise on farms, because they failed to adapt to local socioecological settings, which are usually very different than the on-station biophysical conditions (Sullivan, 2001; Mekoya et al., 2008). This explains why farmers/pastoralists have very different forage preferences relative to recommendations of forage agronomists and animal nutritionists (Mekoya et al., 2008; Balehegn et al., 2015).

Weak extension systems also limit adoption of feed technologies and this may be considered among the main constraints. (Kebebe, 2019). In many feed development projects, sustained extension support beyond life of research projects is completely lacking in most LMIC (Shapiro et al., 2015). Where they exist, extension services in many LMIC focus on crop production, which is usually prioritized to achieve human food security at the expense of livestock production. For instance, in many rural areas in Ethiopia, livestock development agents are required to engage in months of extensive crop production activities thus abandoning support for the livestock production sector (Tolera et al., 2012).

**Fig. 1:**
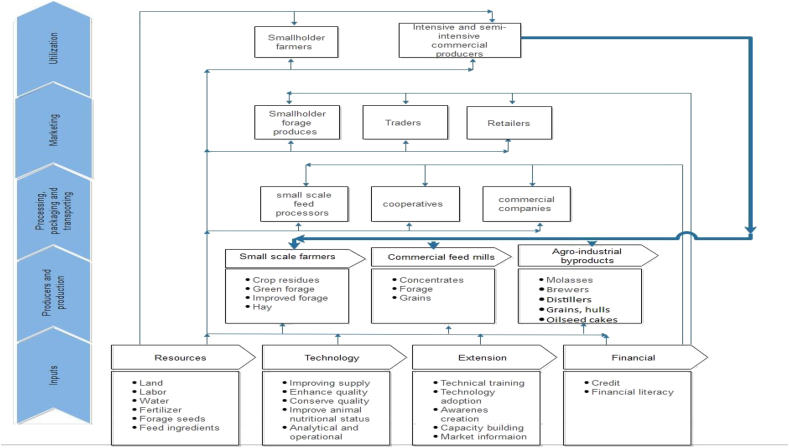
A typical feed value chain in low- and middle-income countries (thin arrows show weaker links and the thick bold arrow shows the only strong link, i.e., high demand for all types of feeds from intensive and semi-intensive commercial producers).

Underdeveloped feed value chains dominated by artisanal and small-scale producers (Ayantunde et al., 2014) are also important constraints to feed development in LMIC. Fig. 1 shows a typical LMIC feed value chain with emphasis on the weakest linkages that need development and the strongest ones that need to be enhanced. The feed value chains in LMIC are characterized by: limited capacity to produce feeds and feed ingredients (Van der lee et al., 2014) and exploit the production capacity of feed mills; marked seasonal and other fluctuations in ingredient supply and quality (Singh et al., 2013; Lukuyu et al., 2016); supply logistics challenges particularly for bulky crop residues; and absence of market incentives for improving quality such that prices are unrelated to quality. Additional factors include absence of vibrant seed systems for forage species; weak governance of the value chain; absence of market information systems; and absence of enabling policies (Konlan et al., 2018). In many LMIC, quality and safety regulatory institutions also either do not exist or lack the means or authority to incentivize or deploy penalties. Consequently, there is limited awareness among smallholders about the value of feed quality and safety regulation (Tolera et al., 2012). Yet a well-developed feed value chain with active participation of the private sector is critical for encouraging innovation and adoption of technologies in the feed sub-sector.

The aforementioned highlights the fact that development and introduction of feed improvement technologies alone are not enough. Rather, it is critical to also understand the contextual factors that facilitate or hinder their uptake in specific communities (Kebebe, 2019). Approaches that recognize and address the multiple and varied objectives of smallholders and the associated tradeoffs are required for successful implementation of livestock development projects (Salmon et al., 2018). Holistic understanding of technology adoption by smallholder famers requires a shift from the household characteristics-based understanding of technology adoption to considerations of factors affecting adoption at different aggregation levels including the farm household, value chain, institution and national policy (Bergek et al., 2015).

## 2 Successful feed enhancement interventions and underlying factors

Despite the challenges described above, several introduced feed technologies have improved supply of quality feed and livestock productivity and have been successfully adopted, scaled and some have directly increased incomes (White et al., 2013). Examples include brown midrib sorghum in central America (Rodriguez, 2013), *Desho* grass in Ethiopia (Asmare et al., 2016), *Brachiaria* in Brazil and Kenya (Jank et al., 2014; Maina et al., 2019), cowpea in West Africa (Tarawali et al., 2002), corn silage production in semi-arid China (Gansu Economic Daily, 2018) and *Ficus thonninningii* trees in northern Ethiopia (Balehegn et al., 2014a). Table 3 describes some successful feed improvement technologies in various LMIC and agro-ecologies.

**Table 3:** Successful interventions that improved feed quality and supply in low- and middle-income countries.

Category	Technology	Country and agro-ecology	Description of intervention(s)	Description of results and impacts	Reference
Feed quality enhancement	Concentrate feed and urea-treated sorghum stover supplementation	India	Supplementation with locally available nutritionally dense supplementary feeds such as maize grains	Average milk yield increased by 1.25 liters per day; farmers' income increased by 34 Indian rupees (~50 US cents) per day per animal	ILRI Asia, 2015
	Urea- ammonia treatment of crop residues	China, India	Treatment of lignified crop residues using urea-ammonia solution to improve their CP content, digestibility and intake	Large scale utilization of crop residues in countries where it has been adopted	Owen et al., 2012
	Reducing particle size of crop residues	India, Pakistan	Techniques such as chaffing, chopping, shredding, pulverizing, grinding are applied to reduce the particle size of crop residues, mainly stovers	Reduced ruminal retention time and thus improved voluntary feed intake partly due to easier mixing of ground crop residues with concentrates, improving crop residue utilization.	Owen et al., 2012
Feed productivity improvement	Introduction of improved forage plants	Ethiopia	Planting forages in backyard, intercropping, integrating forage plants with soil and water conservation in degraded areas, agro-forestry, over-sowing, etc.	Increased forage yield from 282 metric tons in 2016 to 468 metric tons in 2017	ACDI-VOCA, 2019
	Irrigated forage production	Ethiopia	Production of supplemental green fodder using irrigation in smallholder farming systems in the highlands of Ethiopia	Milk yield increased from 2.3 liters to 4.6 liters/day/local cow, translating into an income growth of USD 135–170/month for farmers owning 3–4 crossbred cows. Number of adopters grew from 17 farmers in 2017 to 500 in 2019	ILRI-LIVES 2014; Karaimu, 2019
	Silvopastoral production	Ethiopia/degraded arid and semi-arid areas	Planting of locally available trees and shrubs that produce nutritious fodder in pasturelands, farmlands, degraded areas, etc.	Improved nutritional status of animals e.g. replacing 50% of commercial concentrates with leaf meal improved goat productivity; technology adopted by more than 20,000 new households; contributed to rehabilitation of degraded grazing lands	Balehegn et al., 2014a
	Desho grass (*Pennisetum pedicellatum*)	Ethiopia	A local, drought tolerant and high biomass production cultivar of *Pennisetum pedicellatum*, distributed through farmer to farmer experience sharing and innovation	Improved forage productivity with some farmers producing up to 61,890 kg DM per year of forage	Shiferaw et al., 2011
	Brown midrib sorghum and maize varieties	Central and south America	Sorghum, maize and millet sorghum cultivars that uniquely combine agronomic adaptation, high biomass and grain yield as well as forage quality	Brown midrib forage resulted in an average increase of milk production of 1.64 kg per day in seven studies	Sánchez-Duarte et al., 2019
	Cultivation of Cowpea	West Africa	A multi-disciplinary and multi-center approach to working with farmers which combines complementary strengths of different actors including international and national research institutions.	The approach resulted in extensive adoption cow pea as a dual-purpose forage crop among commercial and small-scale livestock producers.	Tarawali et al., 2002 and Singh et al., 2003
	Fodder tree legumes	Kenya	The use of various strategies (Intercropping, on back yards, conservation areas, under irrigation) for planting forage tree legumes.	Success was a result of building partnerships with diverse stakeholders, ensuring appropriateness of practice, assisting local communities to mobilize resource and ensuring participation of farmers in evaluation of practices	Franzel et al., 2003
	Cactus	Algeria	Planting cactus in degraded natural rangelands and using cactus to feed cattle	Planting cactus as forage resulted in 57.6 times higher carrying capacity than native dryland rangeland	Koeleman, 2015
	Cultivation of *Brachiaria*	Brazil	Cultivation of different Brachiaria species in low fertility status soils	Most *Brachiaria* varieties adapt well to acidic, low-fertility soils. As a result, an estimated 99 million hectares of *Brachiaria* species have been sown as improved pastures in Brazil. In Kenya, adoption of *Brachiaria* has resulted in a 27.6% increase in milk yield among small scale dairy producers.	Jank et al., 2014; Maina et al., 2019
	Exclosure	Ethiopia/degraded arid and semi-arid areas	Fallowing degraded rangelands to allow natural rehabilitation and increase pastureland productivity	Increased forage biomass (by more than 150%), for livestock and improved carrying capacity of rangelands	Yayneshet et al., 2009; Wang et al., 2019
Enhancement of the nutritional status of animals	Urea molasses multi-nutrient block	India	Providing multi-nutrient blocks to animals on low quality basal diets	Improved crop-residue intake, reproductive efficiency and milk yield	Owen et al., 2012
Feed quality maintenance or preservation	Silage making	China	Government, farmer, industry, financial and extension sector integrated approach for introduction of silage making in the semi-arid and hilly region of Loess Plateau, China	Increased household income by 28.6% between 2010 and 2017 and increased meat production by 48.3% between 2013 and 2018	Gansu Economic Daily, 2018
	Silage and Saltlick blocks	Burkina Faso	Sprinkling salt in the herbage during silage production produces salt-laden soil as a by-product, which is used to make saltlick blocks	Resulted in cost benefit ratio of a cost-of 527%, and extensively adopted by farmers not just for feeding animals, but also making profits from sold forage. Between year 2002 and 2003, the numbers of beneficiary farmers increased from 120 to 537 farmers. The technology has now been spread to 12 groups in 17 villages, each consisting of 50–80 farmers	Kassam et al., 2009
Analytical and operational technologies	Mobile app feeding tool to optimize milk production	Nepal	Mobile app-based feeding support tool which enables formulation of least cost, nutritionally balanced rations for dairy cattle and buffalo and prediction of milk yield	95% of dairy farmers (n = 55) who used the new tool reported an increase in milk yield by an average of 1 l daily. This technology is currently being scaled to 1600 dairy cooperatives in Nepal	Shrestha, 2019
	Development of urban and peri-urban fodder markets	India, Tanzania	Growing, collecting and buying fodder to sell to urban and peri-urban dairy and fattening farmers	These have been encouraged by the increased livestock feed demand in urban and peri-urban farms.	Singh et al., 2013; Lukuyu et al., 2016

Most of the successes did not depend on the nature of technologies *per se*, but on specific local conditions that facilitated their adoption by farmers (Gebremedhin et al., 2003a). For instance, successful adoption of forage legumes depends on their ability to meet farmers’ needs, building relevant partnerships, understand the socio-economic context and skills of famers and participatory involvement of communities, particularly champions (Shelton et al., 2005). That success is not based just on the technology is evident because technologies, which have not been adopted in sub-Saharan Africa, e.g. crop residue ammoniation, have been adopted in China and to a lesser extent in India (Owen et al., 2012). Technologies such as urea treatment of crop residues work only when they are properly implemented (Owen et al., 2012; Salem and Smith, 2008) and when adequate resources, infrastructure and technical skills are available for their use in smaller scale production systems. Thus, feed development interventions that succeed are those that focus on technologies that are good fits for the prevailing socio-economic and cultural settings. Consequently, to facilitate adoption, participatory technology development should be coupled with extension efforts that recognize agro-ecological and socio-economic contexts as well as appreciating and incorporating knowledge from various sources, rather than from scientists or researchers alone (Leeuwis and Aarts, 2011; Conroy, 2005). Moreover, due to the multifaceted nature of feed challenges in LMIC, feed technologies that deliver multiple benefits are often more successful. An enabling environment that supports and or rewards technology adoption by farmers is also an important prerequisite for success (Van Huis et al., 2007).

Further, success has also resulted from adoption of a combination of technologies (package-approach) that result in synergistic improvements in profits such as providing improved feeds to high genetic merit livestock breeds with greater performance potential or improving capacity in feed quality analysis and marketing (Redjal, 2005). Additional examples are improved forage introduction and silvopasture in semi-arid Ethiopia (Balehegn et al., 2014a), which provided simultaneous solutions to different challenges including feed scarcity, land degradation, and lack of fuel wood, or the dual-purpose (food and feed) brown midrib sorghum variety in South America (Sánchez-Duarte et al., 2019), which provided more digestible stover for animal feed as well as grain for human consumption. Such approaches require proper evaluation of technologies and their fit to given social and agroecological systems from the outset. The Livestock Systems Innovation Lab EQUIP-FEED project follows this package or holistic approach to try to solve the livestock feed problems in Ethiopia and Burkina Faso (see http://livestocklab.ifas.ufl.edu/projects/feed-project). The project has five components across the feed value chain and aims to develop the knowledge, skills, tools and products in the production, processing and utilization of feed towards an eventual increase in the supply of quality feed in Burkina Faso and Ethiopia. The five components aim to implement important solutions across the feed value chain namely; 1: Understanding available feed resources and their challenges; 2. Develop best-bet forage options that are adapted to various agro-ecologies; 3. Develop more accurate nutrient requirement values for local and cross-bred animals and develop rations that are better nutritionally and cost wise.; 4. Improve the capacity for analysis and quality standardization for feeds to improve commercialization of the feed sector; and 5. Demonstrate the synergistic effect of improved feeding, dairy management and breeding on dairy productivity.

Growing market oriented urban and peri-urban dairy and fattening systems in towns all over sub Saharan-Africa and South East Asia (Alarcon et al., 2017) are also associated with increased demand for feed. As a result, small-scale fodder marketing and growing of high yielding forage cultivars is increasing (Ponnusamy et al., 2017). While most fodder sellers are small scale producers who produce more feed than they need for their own animals (Lukuyu et al., 2016), there is a continuously growing demand for fodder markets from urban and peri-urban commercial livestock producers (Singh et al., 2013).

## 3 Conclusions and implications

Limited supply of quality feed is the main constraint to development of the livestock sector in many LMIC, and it constrains attainment of food and nutritional security. Despite the wealth of ‘research-proven’ technologies that can be used to improve feed and hence livestock production in smallholder systems in such countries, only a few success stories exist because of the low level of adoption of the “promising” technologies by the farmers.

The failures of adoption of feed improvement technologies result from systemic constraints that make their adoption challenging and from paying inadequate attention to sociocultural and economic norms. Even when technical and resource limitations are addressed, the limited scale of improvement in livestock productivity from some technologies may not adequately incentivize adoption of the technology.

Where success stories with widespread adoption of technologies exist, they are often driven by financial and market incentives and or by simultaneous provision of solutions to different problems while addressing socioeconomic factors. Such examples typically require collaboration between research, extension and financial institutions. Therefore, participatory technology development involving various key stakeholders (farmers, extension, financial institutions, the private sector) is a promising approach. While it is important to increase the diversity of potentially appropriate ‘working technologies’ that target specific agro-ecologies and production systems, it is also critical to understand their fit to the specific context and to ensure that the enabling environment exists. The Techfit tool (now redesigned as the Intervention Ranking Analysis Tool in FEAST) (Duncan et al., 2012), for example, attempts to match technologies to local conditions considering important context-specific constraints. Such tools help researchers to think through the characteristics of the local system including the prevailing sociocultural and other norms, and thus select those that are most likely to be widely adopted.

Feed-related constraints are only a subset of the range of challenges faced by smallholder farmers. Other overriding challenges should be considered such as lack of market access for selling livestock or their products, lack of finances, low genetic merit livestock breeds that inadequately respond to improved feeding, and diseases that limit animal productivity. Therefore, a ‘package approach’ that improves various production aspects and or various components of the value chain is more likely to be successfully adopted. It is also critical to ensure private sector engagement from the outset to ensure sustainability and scaling of the intervention after donor or research funding ends.

Finally, given the complexity of the problem of adoption of feed technologies by smallholders, future research in the livestock sector should shift from developing new technologies towards assessing socio-cultural and institutional barriers to adoption of technologies and finding innovative ways of bypassing such barriers (Kebebe, 2019). This entails a shift from the bio-physical focus to developing alternative institutional arrangements that improve engagement of stakeholders, including farmers, the private sector and strengthening of the value chain (Peters et al., 2001; Lenné et al., 2003; Kebebe, 2019). There is also an urgent need for prioritizing and reforming specific regulations and policies that currently deter or limit private sector investment in small- and medium-sized agribusinesses in the feed value chain. Collectively, these approaches will facilitate adoption of feed technologies, improve livestock productivity and contribute to reducing food and nutrition insecurity problems in LMIC.

## Declaration of competing interest

The authors declare that there is no any conflict of interest.
